# Age-related hearing impairment and frailty in Alzheimer's disease: interconnected associations and mechanisms

**DOI:** 10.3389/fnagi.2015.00113

**Published:** 2015-06-09

**Authors:** Francesco Panza, Vincenzo Solfrizzi, Davide Seripa, Bruno P. Imbimbo, Rosa Capozzo, Nicola Quaranta, Alberto Pilotto, Giancarlo Logroscino

**Affiliations:** ^1^Neurodegenerative Disease Unit, Department of Basic Medicine, Neuroscience, and Sense Organs, University of Bari Aldo MoroBari, Italy; ^2^Department of Clinical Research in Neurology, University of Bari Aldo Moro, “Pia Fondazione Cardinale G. Panico”Tricase, Italy; ^3^Geriatric Unit, Laboratory of Gerontology and Geriatrics, Department of Medical Sciences, IRCCS “Casa Sollievo della Sofferenza”San Giovanni Rotondo, Italy; ^4^Geriatric Medicine-Memory Unit and Rare Disease Centre, Interdisciplinary Medicine Department, University of Bari Aldo MoroBari, Italy; ^5^Research and Development DepartmentChiesi Farmaceutici, Parma, Italy; ^6^Otolaryngology Unit, Department of Basic Medical Science, Neuroscience and Sensory Organs, University of Bari Aldo MoroBari, Italy; ^7^Geriatrics Unit, Department of OrthoGeriatrics, Rehabilitation and Stabilitation, Frailty Area, Galliera Hospital NR-HSGenova, Italy

**Keywords:** Alzheimer's disease, dementia, mild cognitive impairment, age-related hearing impairment, peripheral auditory dysfunction, central auditory processing deficit, frailty syndrome

Among potentially modifiable age-related conditions linked to dementia, Alzheimer's disease (AD), and late-life cognitive disorders, age-related hearing impairment (ARHI) or presbycusis is the most widely diffused sensory disorder and one of the principal causes of chronic disability in older adults (Gates and Mills, 2005). The impairments of peripheral (sensory or strial) and central (predominantly neural) auditory pathways, diagnosed with different procedures, are often variously imbricated in determining ARHI, with mixed clinical findings (Gates and Mills, 2005). A growing body of epidemiological evidence linking ARHI with late-life cognitive disorders (Panza et al., 2015a) suggested the potential for correcting hearing loss so that elders can function better also from a cognitive point of view with appropriate treatment.

ARHI is also a substantial marker for frailty in older age, another age-related clinical condition for identifying older persons at elevated risk for numerous adverse health outcomes such as falls, institutionalization, hospitalization, disability, and death (Rodríguez-Mañas, 2013). Frailty is as a multidimensional syndrome characterized by a nonspecific state of vulnerability, reduced multisystem physiological reserve, and decreased resistance to stressors (Rodríguez-Mañas, 2013). Although there is no consensus regarding the operational definition of frailty, in general, two are the most frequently used approaches: the first is the physical or “phenotypic” model of frailty, while the second is based on deficit accumulation, measured with the so called frailty indexes, and defined as an accumulation of health-related deficits and disorders (Rodríguez-Mañas, 2013). However, also psychological, cognitive and social factors are part of this multidimensional syndrome, with great influence on its definition and treatment. Cognition has already been suggested as a possible component of frailty with increased risk of adverse outcomes. Therefore, the prevention of cognitive-related adverse outcomes including delirium (Eeles et al., 2012) and late-life cognitive disorders (Robertson et al., 2013; Panza et al., 2015b) may be possible also through frailty prevention.

## Peripheral age-related hearing impairment, Alzheimer's disease, and cognition

In the USA, ARHI is even more prevalent among older adults than previously reported, with the ARHI prevalence that approximately doubles every decade of life from the second through to the seventh decade (Quaranta et al., 2015). At early stage, ARHI typically affects audibility of the higher frequencies (6000 and 8000 Hz), interfering with regular speech comprehension in both quiet and noise, and spreading to the mid and low frequencies over time. Part of the hearing problems are not related to the peripheral deficit of the auditory system but to the central auditory processing (CAP) dysfunction. Subjects with this condition have considerable difficulty in understanding speech in presence of a background noise or in reverberant rooms, but no problem in a quiet environment. Both peripheral and central auditory dysfunctions are therefore relevant to assess a possible influence of ARHI on late-life cognitive disorders.

Over two decades ago, a review article with also original findings summarized the first 25 years of research on possible associations between hearing impairment and cognitive dysfunction reviewing a relevant number of reports (Gennis et al., 1991). The cumulative evidence coming from this first series of studies suggested a strong link between peripheral ARHI and cognitive impairment or decline in demented or institutionalized patients, while there was no association in nondemented older subjects (Gennis et al., 1991). More recently, a case-control report on AD patients from a tertiary center (Gold et al., 1996) and a number of cross-sectional (Gussekloo et al., 2005; Lin, 2011) and longitudinal population-based studies (Lin et al., 2004, 2011, 2013; Valentijn et al., 2005; Wallhagen et al., 2008; Gallacher et al., 2012; Kiely et al., 2012) (Table 1) confirmed the interplay among peripheral auditory dysfunction and AD, dementia, and cognitive disorders in older age, with a single exception in a 2-year follow-up of the Australian Longitudinal Study of Aging (Anstey et al., 2001). However, a weak association between hearing loss and memory decline was found in a re-evaluation of the same sample in a longer follow-up of 8 years (Anstey et al., 2003) (Table 1).

**Table 1:** **Principal case-control and longitudinal population-based studies of peripheral auditory dysfunction and central auditory processing dysfunction in age-related hearing impairment in relation to late-life cognitive decline, incident dementia and Alzheimer's disease (AD)**.

**References**	**Subjects**	**Assessment of auditory function**	**Assessment of cognition or diagnosis of dementia**	**Covariates**	**Principal findings**
**PERIPHERAL AUDITORY DYSFUNCTION**					
Anstey et al., 2001 The Australian Longitudinal Study of Aging Australia	2087 subjects aged 70–96 years	Pure-tone audiometry with portable audiometers	Similarities and DSS from WAIS-R, BNT, and NART	Age	In a 2-year follow-up, decline in hearing was not associated with decline in any cognitive domain
Anstey et al., 2003 The Australian Longitudinal Study of Aging Australia	525 subjects, mean age 74.7 years	Pure-tone audiometry with portable audiometers	Similarities and DSS from WAIS-R, BNT, and NART	Age, gender, depressive symptoms, self-rated health, and medical comorbidities	In a 8-year follow-up, a weak association between hearing decline and memory decline was found
Lin et al., 2004 The Study of Osteoporotic Fractures (SOF) USA	6112 women aged 69 years and older	Pure-tone audiometry with hand-held audiometers	3MS and functional status assessed with five activities: walking, climbing stairs, preparing meals, shopping, and doing housework	Medical comorbidities, age, education level, smoking, presence of vertebral fracture, benzodiazepine use, BMI, Lubben social network, grip strength, walking speed, and baseline cognitive or functional status	Combined hearing and vision impairment was associated with cognitive and functional decline after a 4-year follow-up period
Valentijn et al., 2005 The Maastricht Aging Study The Netherlands	418 subjects aged 55 years and older	Pure-tone audiometry	VVLT, SCWT, CST, VFT, and LDST	Age, sex, education level, baseline cognitive performance, change in vision or hearing, and baseline vision or hearing	A change in auditory acuity predicted change in memory performance, and auditory acuity at baseline predicted change in the mean score of the first two SCWT cards and the LDST in a 6-year follow-up
Wallhagen et al., 2008 The Alameda County Study USA	2002 subjects aged 50–94 years	Self-report	A self-report measure of cognitive functioning	Chronic conditions, age, sex, and education level	In a 5-year follow-up, a relationship between baseline hearing impairment and subsequent poorer cognitive function was found in both existing and new cases of cognitive impairment
Lin et al., 2011 The Baltimore Longitudinal Study of Aging USA	639 subjects aged 36–90 years	Pure-tone audiometry	Diagnoses of dementia and AD using DSM-III-R and NINCDS-ADRDA criteria	Sex, age, race, education level, diabetes, smoking, hypertension, and hearing aid use	During a median follow-up of 11.9 years, baseline hearing loss was independently associated with incident all-cause dementia
Gallacher et al., 2012 The Caerphilly cohort United Kingdom	1057 men mean age 56.2 years	Pure-tone audiometry.	Diagnoses of dementia and AD using DSM-IV and NINCDS-ADRDA criteria. MMSE, CAMCOG, AH4, and CRT	Age, social class, anxiety, and premorbid cognitive ability	Over a 17-year period, auditory threshold was found to be associated with incident dementia and cognitive decline. An additional effect of change in auditory threshold over 8 years was found for nonvascular dementia
Kiely et al., 2012 The Dynamic Analyses to Optimize Aging Project Australia	4221 subjects aged 50–103 years	Pure-tone audiometry.	MMSE	Age, sex, diabetes, stroke, hypertension, visual impairment, smoking status, workplace noise exposure, and high-frequency audiometric noise notches	Cognitive impairment and hypertension were independently associated with lower levels and accelerated declines of peripheral hearing, and incidence of cognitive impairment was also associated with poorer hearing function in a 11-year follow-up
Lin et al., 2013 The Health ABC Study USA	1984 subjects mean age: 77.4 Years	Pure-tone audiometry.	3MS and DSS	Age, sex, race/ethnicity, education level, study site, smoking status, hypertension, diabetes mellitus, and stroke history	In a 6-year follow-up, hearing loss was independently associated with accelerated cognitive decline and incident cognitive impairment
**CENTRAL AUDITORY PROCESSING DYSFUNCTION**					
Gennis et al., 1991 USA	112 community-dwelling adults aged 60 years and older	Pure-tone audiometry and SPIN	WMS and JCST	Age and sex	In a 5-years follow-up, no evidence that peripheral or central hearing impairment predicted cognitive decline was found
Gates et al., 1996 Framingham Heart study USA	Population-based, 1662 people aged 63–95 years	Pure-tone audiometry and SSI-ICM	MMSE and diagnosis of dementia and AD using NINCDS-ADRDA criteria	Age and education level	In a 6-year follow-up, CAP dysfunction in one ear increased the risk of subsequent dementia or cognitive decline; CAP deficit present in both ears doubled the risk
Gates et al., 2002 Framingham Heart study USA	Population-based, 740 people aged 63–95 years	Pure-tone audiometry and SSI-ICM	MMSE and diagnosis of probable AD using NINCDS-ADRDA criteria	Age, gender, education level, APOE e4 allele presence, and hearing level	In a 8-year follow-up, CAP dysfunction presence was associated with a 10 times higher risk for developing AD
Gates et al., 2011 The Adult Changes in Thought Study USA	Population-based, 274 people aged 71–96 years	Pure-tone audiometry, SSI-ICM, DSI test, and DDT	CASI and diagnoses of dementia and AD using DSM-IV and NINCDS-ADRDA criteria	Education level	In a 3-year follow-up, severe CAP dysfunction strongly predicted the risk of a subsequent diagnosis of AD
Idrizbegovic et al., 2013 Sweden	Case-control, 70 participants aged 50–80 years with MCI or AD and SMC	Pure-tone audiometry, SPIN, and DDT	MMSE, MCI diagnosis, and diagnoses of dementia and AD using DSM-IV and NINCDS-ADRDA criteria	None	In a 1.5-year follow-up, CAP showed a significant decline in the AD group compared with the controls/SMC subjects (left ear)

*DSS, Digit Symbol Substitution subscale; WAIS-R, Wechsler Adult Intelligence Scale-revised; BNT, Boston Naming Test; NART, National Adult Reading Test; 3MS, modified version of Mini-Mental State Examination; BMI, body mass index; VVLT, Visual Verbal Learning Test; SCWT, Stroop Color Word Test; CST, Concept Shifting Task; VFT, Verbal Fluency Test; LDST, Letter-Digit Substitution Test; DSM-III-R, Diagnostic and Statistical Manual of Mental Disorders, third edition, revised; NINCDS-ADRDA, National Institute of Neurological and Communicative Disorders and Stroke and the Alzheimer's Disease and Related Disorders Association; MMSE, Mini-Mental State Examination; DSM-IV, Diagnostic and Statistical Manual of Mental Disorders, Fourth Edition; CAMCOG, Cambridge Cognitive Examination; AH4, Alice Heim test; CRT, 4-choice reaction time task; SPIN, Speech perception in noise; WMS, Wechsler Memory Scale; JCST, Jacobs Cognitive Screening Test; SSI-ICM, Synthetic sentence identification with either an ipsilateral competing message; APOE, apolipoprotein E; DSI, dichotic sentence identification; DDT, dichotic digits test; CASI, Cognitive Abilities Screening Instrument; DSM-IV, Diagnostic and Statistical Manual of Mental Disorders, Fourth Edition; MCI, mild cognitive impairment; SMC, subjective memory complaints*.

## Central auditory processing dysfunction, mild cognitive impairment, and Alzheimer's disease

Age-related decline in CAP appeared not to be an isolated condition, but an entity with a multifactorial nature associated to age- and/or disease-related brain and auditory changes (Gates and Mills, 2005). In fact, CAP dysfunction increases with age (Gates and Mills, 2005), but given the increased incidence of both peripheral ARHI and cognitive impairment in late life, the interpretation of central auditory tests may be difficult. In particular, in the early phase of cognitive decline, the differential impact of the two types of auditory deficits on late-life cognitive disorders may be not easy to determine. Several existing studies mostly focused on the impact of peripheral auditory deficit on late-life cognition, while studies on the link between CAP dysfunction and mild cognitive impairment (MCI) or AD are sparse (Gennis et al., 1991; Kurylo et al., 1993; Gates et al., 1996, 2002, 2008, 2010, 2011; Idrizbegovic et al., 2011, 2013).

Several cross-sectional case-control (Kurylo et al., 1993; Idrizbegovic et al., 2011) and two population-based studies (Gates et al., 2008, 2010) suggested a strong involvement of CAP dysfunction in MCI (Idrizbegovic et al., 2011), dementia (Gates et al., 2010), and AD (Kurylo et al., 1993; Gates et al., 2008; Idrizbegovic et al., 2011). In particular, findings from the Adult Changes in Thought (ACT) Study suggested that CAP disorders may be associated with executive dysfunction in older subject with and without memory loss and dementia (Gates et al., 2010). Therefore, executive control function may be a key factor in the speech-based behavioral tasks evaluating CAP dysfunction because lesions of the central auditory pathway are infrequent in early AD (Kurylo et al., 1993).

A few longitudinal case-control (Idrizbegovic et al., 2013) and population-based studies (Gates et al., 1996, 2002, 2011) suggested that CAP dysfunction in ARHI may be central in determining an increased risk of cognitive decline and incident dementia or AD (Gates et al., 1996), and AD (Gates et al., 2002, 2011) (Table 1). Therefore, deficit in CAP could be an early marker of MCI or AD, with a “gradient” existing in CAP disorders among subjects with subjective memory complaints, MCI, and early AD (Idrizbegovic et al., 2011).

## Age-related hearing impairment-dementia link and frailty

Some factors may be involved in causal mechanistic pathways linking ARHI and cognition, while other factors may constitute shared pathological processes or etiological pathways underlying both ARHI and cognitive disorders in late life. Cognitive testing may be confounded by ARHI in association with poor verbal communication. However, some studies used also nonverbal cognitive tests or were insensitive to the exclusion of subjects with serious hearing loss from the analyses (Anstey et al., 2003; Lin et al., 2013), so not supporting the hypothesis that miscommunication in hearing loss should impair cognitive testing. Furthermore, in older subjects with subclinical cognitive impairment may occur an overdiagnosis of peripheral ARHI, although audiometric testing appeared to be reliable also in patients with early dementia.

Evidence coming from epidemiological (Fratiglioni et al., 2000; Wilson et al., 2007) and neuropathological studies (Bennett et al., 2006) suggested that social isolation and loneliness, caused by communication impairments in older subjects with ARHI, may lead to cognitive decline and AD. Moreover, also the cognitive reserve concept, often conditioned by communication defects, may account for the link between ARHI and cognition. Cognitive/brain reserve appears to be a buffer against the functional impairments caused by accumulating age-related brain changes or AD-related pathology, so acting as a modulator of the interplay between neuropathology and cognitive outcomes. Increased cognitive load to help compensate auditory processing may reduce the neural resources available to other cognitive processes such as working memory and perceptual speed, increasing the deleterious effects of AD pathology and revealing the earlier clinical symptoms of dementia (Boyle et al., 2008). Furthermore, a key link between communication difficulties, social isolation, and cognitive decline is the reduced capacity to participate in mentally stimulating activities. In fact, cognitive reserve acts as a buffer via engaging in cognitively stimulating behaviors within an enriched environment, so enhancing neuroplasticity, Furthermore, while AD-related neuropathology is absent in the peripheral auditory pathways (Sinha et al., 1993), peripheral ARHI may contribute to loss of gray matter volume in primary auditory cortex (Peelle et al., 2011), accelerated rates of decline in regional brain volumes in the right temporal lobe and whole brain volume (Lin et al., 2014), and variation in the integrity of central auditory white matter tracks (Chang et al., 2004). AD-related neurodegeneration may be also involved in peculiar damage of central auditory nuclei required for higher-order auditory processing (Parvizi et al., 2001). However, more serious effects may be caused by damage to higher-order cortical areas involved in language processing (Kurylo et al., 1993), so not excluding a shared neuropathological origin underlying both ARHI and AD/dementia.

Among possible confounders, common pathological processes, or shared etiological pathways linking ARHI and cognition, frailty syndrome could have a central role. In fact, ARHI may be also a strong indicator for both the most widely diffused models of frailty. In some operational definitions and frailty indexes, ARHI is one of the suggested components of frailty (Frailty Index-Comprehensive Geriatric Assessment, Groningen Frailty Indicator, and Puts model) (Panza et al., 2015a) and it may predict functional decline or incident falls in older adults (Lin and Ferrucci, 2012), some of the health-related adverse outcomes linked to frailty. Among potentially modifiable risk factors, there is a growing body of evidence about the impact of several operational definitions of frailty on late-life cognitive disorders (Robertson et al., 2013; Panza et al., 2015b). In particular, an international consensus group has recently proposed the clinical label “cognitive frailty” for describing the simultaneous presence of both physical frailty and cognitive impairment in nondemented older individuals (Kelaiditi et al., 2013), representing also a possible precursor of neurodegenerative processes and AD. Epidemiological studies strongly suggested that physical frailty may be associated with incident AD and MCI, nonAD dementias, vascular dementia (VaD), AD-related neuropathology, and cognitive impairment and decline in late life (Robertson et al., 2013; Solfrizzi et al., 2013; Panza et al., 2015b), so also validating cognitive frailty as a new clinical condition. Several factors and diseases associated with physical frailty are also related to cognitive impairment, including nutritional factors, metabolic disorders, inflammatory markers, hormones, diabetes mellitus, congestive heart failure, and stroke (Robertson et al., 2013; Panza et al., 2015b), suggesting an underlying and shared pathogenesis probably linked to vascular determinants. In fact, in 2006, the term “cognitive frailty” was firstly used to indicate a specific state of cognitive vulnerability in MCI and related entities exposed to vascular risk with a consequent elevated progression to dementia (Panza et al., 2006). Physical frailty has been proposed also as a prodromal stage of VaD (Robertson et al., 2013; Solfrizzi et al., 2013). This could be therefore a further common pathway explaining the ARHI-frailty-cognition interplay given that another neurobiological process such as vascular disease or shared vascular factors may cause ARHI, frailty, and dementia.

## Conclusions and future directions

In recent years, there has been growing attention on the possible correlations between sensorial abnormalities and late-life cognitive disorders. Epidemiological evidence is mainly focused on peripheral auditory disorders and cognition, but in longitudinal population-based studies both peripheral and CAP dysfunctions appear to be associated with incident cognitive impairment and AD and accelerated cognitive decline. While some randomized controlled trials (RCTs) showed improvement in cognitive function or global measures of change in hearing-aid users not cognitively impaired (Mulrow et al., 1990) or with dementia (Allen et al., 2003), determining whether treating hearing loss could delay cognitive decline and dementia remains an open issue. In fact, RCTs with more representative cohorts and technology (i.e., digital hearing aids or cochlear implants), longer follow-up periods, and estimating the effects of hearing rehabilitative interventions on cognitive and global functioning have never been performed (Lin, 2012). At present, use of amplification can be an effective tool for minimizing the perceived disability of older adults and reducing the AD caregiver burden by enhancing the communication abilities of the patients (Palmer et al., 1998). However, hearing aids alone could be not enough to properly manage ARHI, and interventions should be broader, incorporating also concerted counseling, environmental accommodations, and rehabilitative hearing training. Cognition and dementia are causally linked to frailty, and ARHI is also a component of frailty included in several operational definitions. Therefore, frailty could have an important impact in the prevention of late-life cognitive disorders, with nutrition and physical exercise as factors potentially affecting frailty status in advanced age (Clegg et al., 2013). Further investigation on the role of vascular risk on the ARHI-frailty-cognition interplay is warranted to better understand causal mechanisms. Overlaps or interactions among the contributing factors could be investigated supplementing speech-based behavioral measures of CAP with nonbehavioral measures based on electrophysiological studies, and structural, spectroscopic, and functional neuroimaging to detect shared neurobiological markers of ARHI and cognitive decline.

### Conflict of interest statement

The authors declare that the research was conducted in the absence of any commercial or financial relationships that could be construed as a potential conflict of interest.
